# Reducing Sodium Consumption in Mexico: A Strategy to Decrease the Morbidity and Mortality of Cardiovascular Diseases

**DOI:** 10.3389/fpubh.2022.857818

**Published:** 2022-03-22

**Authors:** Ismael Campos-Nonato, Jorge Vargas Meza, Claudia Nieto, Ana Carolina Ariza, Simón Barquera

**Affiliations:** Research Center of Nutrition and Health, National Institute of Public Health, Cuernavaca, Mexico

**Keywords:** sodium, sodium reduction, salt, policy action, public health, Mexico

## Abstract

Hypertension (HTN) and cardiovascular diseases (CVD) are important public health problems in Mexico. High sodium intake is linked to high blood pressure and increased risk of developing CVD. International organizations suggest consuming <2 g of sodium/day; however, the Mexican population consumes amounts above what is recommended: 3.1 g/day. Although efforts have been made to mitigate this problem, interventions are needed to improve cardiovascular health. This policy brief offers a short review of the current sodium consumption situation in Mexico and the importance of why decision makers should consider actions to reduce consumption. Recommendations to reduce sodium/salt intake include: Reformulation of ultra-processed-foods, promote the use warning labels, communication campaign, reduce the use of table salt, and monitor sodium intake.

## Introduction

In Mexico, the main cause of mortality is cardiovascular disease (CVD), with an estimated rate of 134 deaths per 100,000 people (1). High blood pressure, or hypertension (HTN), is the most important risk factor for the development of CVD (2), which two out of four adults experience. This number could be higher, as 55% of people with this disease are often undiagnosed and have no symptoms (3).

One in ten deaths and one in five premature deaths (in people under 70 years of age) are attributable to sodium, both due to cardiovascular causes (4). The World Health Organization (WHO) recommends that sodium consumption does not exceed 2 g per day or 5 g of salt (one teaspoon) (5). However, the Mexican population, including children, adolescents and adults, exceed what is recommended by this organization (6, 7).

Sodium is an essential nutrient to maintain balance in humans since it participates in the regulation of the transmission of nerve impulses, and the contraction or relaxation of skeletal muscle, among other cellular processes (8). However, excess sodium intake causes increased sodium excretion through urine, which induces an increase in vascular resistance and blood pressure or HTN (9). This increase in HTN is related to kidney damage. Likewise, high sodium intake is related to other diseases; for example, to Helicobacter pylori infection, one of the main factors for stomach cancer; increased calcium absorption which leads to the presence of stones; and the consumption of sugary drinks or total energy consumption, which is related to overweight and obesity (4, 9, 10). Therefore, high sodium intake may contribute to poor health (9). In addition, high sodium intake is usually combined with low potassium intake, which is related to different metabolic disorders (11), and increased vascular volume (12).

**Figure 1:**
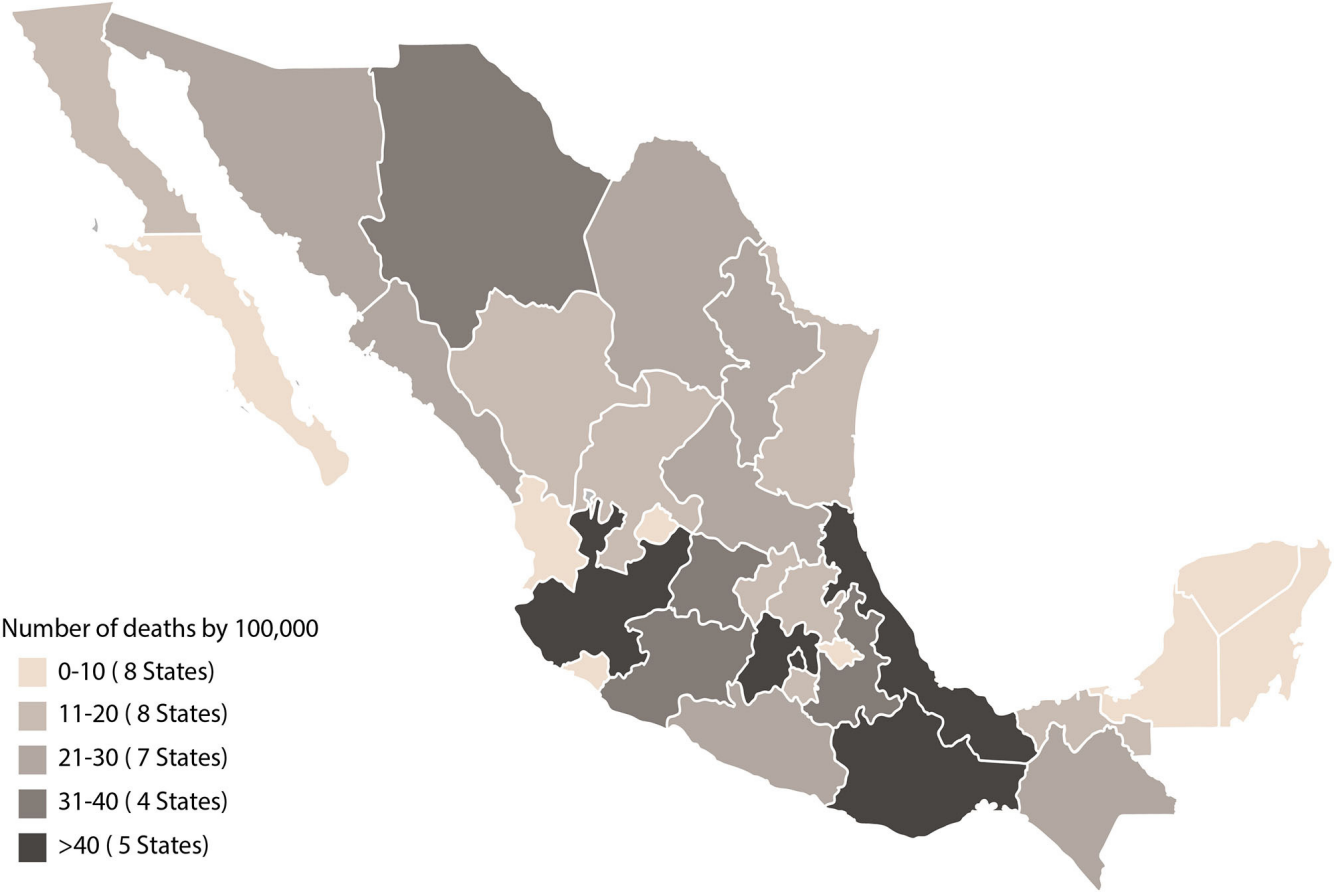
Mortality due to hypertensive causes associated with high sodium intake in Mexico 2019. Source: Global Burden Disease (1).

Of the total deaths in Mexico during 2019 (*n* = 738,424), 23% were due to CVD, of which 5.4% were attributed to high sodium intake. In addition, of the total deaths attributed to high sodium intake, 9% were due to hypertensive disease, 7% to stomach cancer, 5.5% to ischemic heart disease, 5.1% to infarction, and 4.6 % to atrial fibrillation. Among the states with higher mortality from hypertensive disease due to high sodium intake are Oaxaca, Veracruz, Jalisco, State of Mexico, and Mexico City (Figure 1) (1).

Policies to reduce sodium intake are cost-effective to help lower blood pressure (13, 14). Different countries have chosen policies according to their context. The actions that have had the greatest impact are the mandatory reformulation of foods, food labeling, taxes, and communication campaigns, since they have shown greater reductions in the total consumption of sodium in the population (15). This policy brief offers a review of the current sodium consumption situation in Mexico and discusses the importance of considering actions to reduce consumption among the population. It also provides evidence-based actions for decision-makers to reduce sodium intake and improve population cardiovascular health.

## Challenges

### Current Sodium Intake Situation

Sodium is an essential nutrient. Foods and beverages that are naturally high in sodium are milk, eggs, and meat. Sodium is also found in industrialized foods and beverages or in processed and ultra-processed foods, since it is added to enhance flavor and texture, fix colors, and extend shelf life, among other things (16). During the last decade, the purchase of processed and ultra-processed foods has increased among the Mexican population (17), becoming one of the main dietary sources and contributing between 39 and 50% of sodium consumed (6). Ultra-processed foods that contain high added sodium content include: breads, crackers, processed meats, seasonings, and instant soups (18).

However, sodium is not only found in these foods; it is also found in the salt we add in food preparation and in ready-made foods. Due to its use and its importance in the diet, table salt is the second source of sodium in the Mexican diet (6).

In Mexico, most of the population consumes sodium in excess, since school children (5–12 years) consume about 2.8 g of sodium/day (7.1 g salt), adolescents (12–18 years) consume 3.7 g of sodium per day (9.4 g salt), while adults (>18 years) consume 3.1 g of sodium per day (7.8 g salt) (6). In addition, the amount of salt consumed by a large part of the population is still unknown, as well as the adverse health effects of excessive salt intake (19).

**Figure 2:**
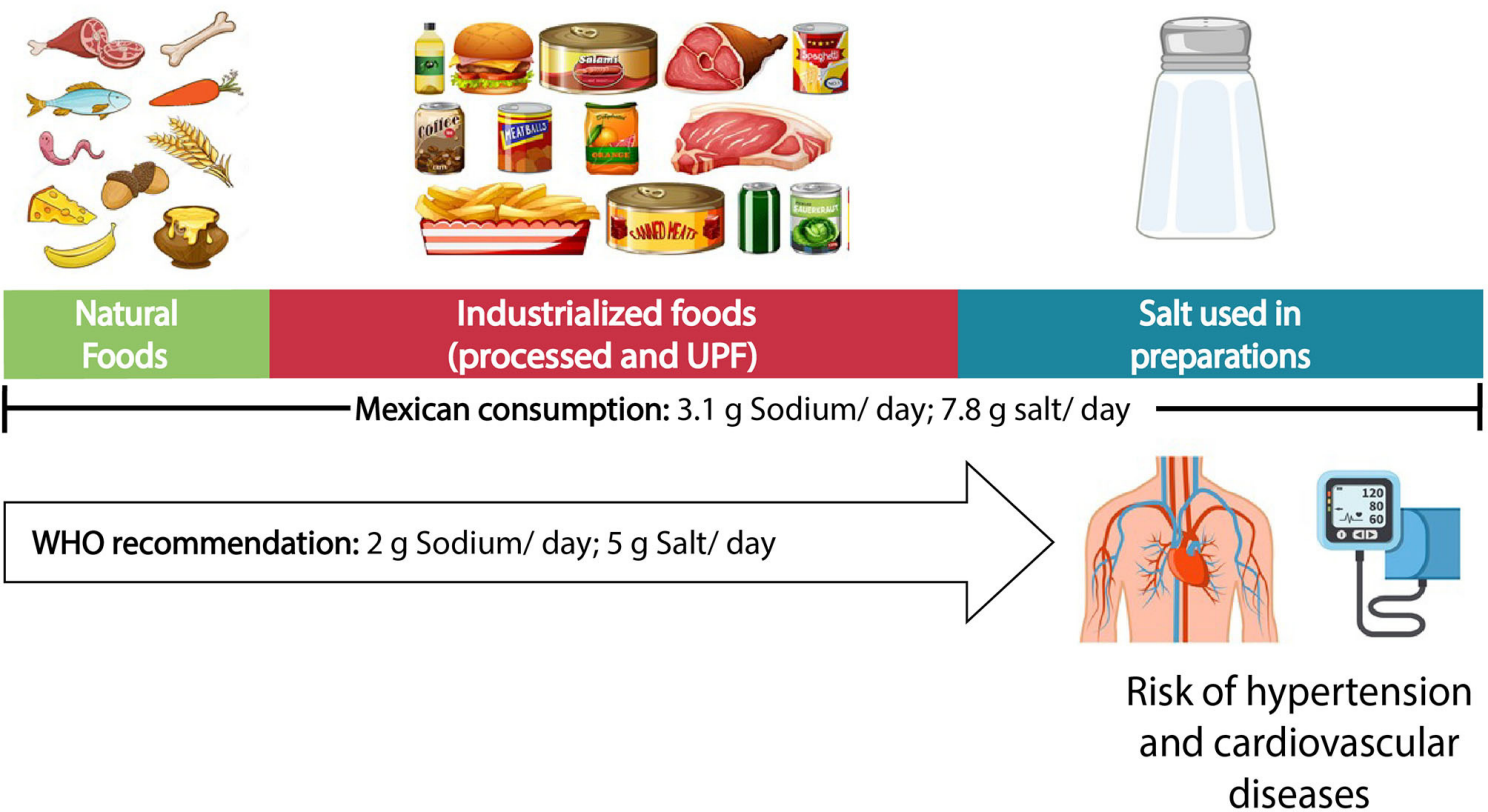
Sources and consumption of sodium in the Mexican population. Source: Own elaboration.

Therefore, daily sodium intake is composed of sodium from natural foods, processed and ultra-processed foods and sodium from salt. However, in Mexico the consumption of this nutrient is above the limits recommended by the WHO (Figure 2). Therefore, the trend of sodium consumption is a public health problem, which, together with the high prevalence of HTN, will affect the presence of complications such as CVD, which could increase mortality rates nationally and should be a priority for the country.

### Actions Carried Out in Latin America to Reduce Sodium Intake

The countries where actions have been implemented to reduce sodium consumption in Latin America. Among the strategies that stand out in the region are, from greater to lesser potential impact, the mandatory reformulation of processed and ultra-processed foods, front-of-pack food labeling systems, healthy eating habits campaigns and the reduction of the use of salt in the production of bread. This impact is similar to the evidence of strategies developed at the international level (15).

In Brazil, where reformulation of processed foods has been carried out, the food industry voluntarily reduced the sodium content of its products (20). Despite the fact that its sustainability could not be ensured due to the results being a voluntary policy, the average sodium content of the included foods decreased from 5 to 21% in these first 2 years of implementation (21).

Other countries, such as Argentina in 2010, have implemented strategies to reduce salt intake. The National Ministry of Health in Argentina launched the “Less Salt, More Life” Initiative to reduce sodium consumption among the general population through different lines of action: voluntary reduction of sodium content in processed foods and community education (22). At the end of 2013, they became the first country in the Americas and the second in the world to implement a mandatory reformulation of sodium content in packaged products (23). Four years after this law went into effect (2014), an evaluation found that over 90% of the products included in the national sodium reduction law met the goals outline in the Standard (24).

On the other hand, Costa Rica developed a Regional Plan for Social Marketing and Communication for the Reduction of Salt in Latin America (25). So far it is unknown what the effects of this intervention are; however, these strategies are recognized by the WHO, due to their potential to increase knowledge to create behavioral changes (26).

To date, eight of the Latin American countries have implemented a mandatory food labeling system to try to reduce the intake of salt / sodium in the population. Five of these countries (Chile, Uruguay, Peru, and Mexico) have used a warning labeling system (27–30), which has been shown to be more effective in promoting healthier habits among Latin American populations and reducing sodium consumption in the population (31). Due to the positive results, other countries such as Brazil and Colombia are in the process of implementing this warning label system (32, 33).

Recently (2021), the WHO (Global and Regional) published updated sodium benchmarks for different food categories, which helps promote the reformulation of food products and make progress in sodium reduction (34, 35). Therefore, it is important to consider these efforts by international agencies, as they are useful for countries in establishing national policies and strategies.

### Strategies Carried Out in Mexico to Reduce Sodium Intake

Despite the fact that non-communicable chronic diseases emerged a little more than two decades ago in Mexico, and that during 2016, two of these diseases were identified as epidemiological emergencies (36), few efforts have been made to treat cardiovascular problems such as HTN and its complications.

One of the most relevant strategies was employed in 2010 during the launch of the National Agreement for Food Health as a strategy against overweight and non-communicable chronic diseases. Some of the objectives of this agreement were to decrease daily sodium consumption; reduce the amount of sodium added to foods; and increase the availability and accessibility of low-sodium or sodium-free products, fostering the participation of different sectors such as the government, civil society, and the food and restaurant industry (37). To meet this objective, the Federal Commission for the Protection against Sanitary Risks implemented the Healthy Mexico Program, targeting restaurants and industrial canteens. This program raised awareness through providing training on healthy eating for the food industry and restaurants. In addition, they encouraged the restaurant community to voluntarily remove salt shakers from the tables and only provide it upon request from the customer (38).

During 2013, the Ministry of Health implemented the Less Salt More Health campaign, which consisted of a communication strategy to increase knowledge and educate the population about the consumption of salt and its benefits (39). However, no publications were identified regarding the results of this strategy.

In March 2020, the modification to the Official Mexican Standard 051 (NOM for its Spanish acronym) was published, which made front of pack warning labels mandatory. This system consists of five octagonal warning labels with the word “EXCESO” (“Excess”) followed by sodium, saturated fat, trans fat, sugar, and calories. It also includes two warning legends for products containing sweeteners and caffeine (30). Given the recent implementation of NOM-051, results from this policy are not yet available. However, Chile, which was the first country to implement a warning labeling system, has documented a reduction in the number of products offered in the market after the implementation of the warning labels, compared to the products that were offered before (31).

## Benefits of Reducing Sodium Intake

The main reason to consider reducing sodium consumption is that it is closely related to blood pressure; as sodium intake increases, blood pressure also increases (40). Therefore, reducing sodium consumption at the population level is one of the most economical and profitable strategies to reduce blood pressure (13), as well as mortality and the risk of developing CVD (41, 42).

On the other hand, studies that simulate the effect on health when sodium consumption is reduced have shown that consuming what is recommended by the WHO (<2 g sodium) reduces systolic/diastolic pressure by 7/4 mmHg in people with HTN and 4/2 mmHg in normotensive people (43). In addition, a study from Brazil has estimated that 47,000 deaths from CVD could be prevented by reducing sodium intake as recommended by the WHO (44). Costa Rica also estimated that 13% of all deaths from cardiovascular causes (*n* = 5,649) could be prevented if current sodium consumption is reduced by 46% (4 g/day), mainly in coronary heart disease and heart disease (45).

Furthermore, a higher threshold (<3 g sodium) has also shown positive effects since it could reduce morbidity from coronary heart disease, stroke and myocardial infarction by 50%, as well as reduce mortality from any cause by 48% (46). Likewise, Brazil has estimated that the health system could save approximately $220 million dollars for the treatment of coronary heart disease and stroke, as well as ~$71 million dollars in indirect costs if the government established voluntary maximum limits for the sodium content on a 20 year plan (2013 to 2032) (47).

## Recommendations

In order to gather the best strategies for reducing sodium consumption in the population, the Pan American Health Organization (PAHO) recently published the guide SHAKE (48). Below are some of the interventions proposed in this publication, highlighting their importance within the Mexican context.

- According to the WHO and the American Heart Association, it is important to reduce sodium intake in adults to <2 g/day (5 g/day of salt) to prevent HTN and the risk of CVD, stroke and coronary heart disease (5, 26, 49).
- The Mexican dietary guidelines should continue to include a recommendation to reduce salt/sodium intake in the first years of life. This will help children learn to enjoy natural flavors and may help them avoid excessive consumption of salt/sodium in the future (50). In addition, the guidelines will support the design of other policies or programs in the country.
- It is important to promote the reduction of table salt used when preparing and consuming food from early life stages, as well avoidance of foods that contain large amounts of sodium, through communication campaigns that encourage behavior change and increase knowledge about health problems associated with high sodium/salt intake. This action is important since part of the Mexican population does not know what their daily sodium /salt consumption should be and is not aware of the health repercussions posed by the high consumption of this nutrient (19).
- Recently, Mexico implemented a mandatory warning labeling system for all available industrialized products (30). It is important to promote the use of this labeling system to reduce the selection and consumption of products that contain excessive amounts of sodium (“Exceso de Sodio”). This action is one of the greatest emphases to be made, since processed and ultra-processed foods are the source that contributes the most sodium to the Mexican diet.
- In addition to the above, it is important to promote reformulation strategies for processed and ultra-processed foods that contain large amounts of sodium. Decreasing the sodium content in these products would reduce the total sodium intake in the population's diet. Although it may be challenging for the food industry to reduce the amount of sodium in their products, some industrialized food producers have been able to do so successfully to avoid “excess sodium” labels on their products (51, 52). However, this reformulation must be in accordance with the WHO regional sodium benchmarks in order to comply with the standards imposed by international agencies (35). Latin American countries have managed to implement this action, including Argentina, Chile, Colombia and Brazil (53–56).
- It is important to encourage the use of salt alternatives added to food to reduce the amount of sodium consumed by the population. Mexico has a great diversity of herbs, spices, and different dried chilis.
- WHO suggests continuous monitoring of sodium intake and sources. They also recommend monitoring and evaluating the programs and actions that have been implemented to reduce sodium. This allows programs to be redesigned, providing evidence for future interventions and scalability.

## Conclusions

Sodium consumption is usually a health problem that is “not visible” and poorly positioned in the public agenda as one of the main risk factors for chronic diseases. However, excessive sodium intake is a public health problem, which is directly related with HTN and CVD.

The actions described above provide the federal government, as well as stakeholders, with public policy tools that help consumers make healthier and more informed decisions to reduce sodium/salt intake. Some successful strategies to reduce sodium consumption in the population have been implemented in: (1) Brazil (voluntary) and Argentina (mandatory), which reduced the sodium content of foods and beverages that contributed to high sodium consumption in the population through reformulation; (2) Chile, Costa Rica, Argentina and Colombia, which all carried out communication campaigns through the “Less Salt more health” strategy; and (3) Argentina, Costa Rica and Chile, which reduced added salt in bread (57). Most of the countries have a comprehensive strategy and have previously carried out more than two actions. However, multiple strategies exist around the world to mitigate this problem; for example, the reduction of the sodium content in food prepared or consumed outside the home; taxes on foods with a high sodium content; regulation of foods in specific settings (schools and hospitals) (15); as well as specific warning messages on salt containers (Argentina is in the process of implementing the latter) (23).

By implementing a set of these interventions with a comprehensive strategy, sodium consumption at the population level could be close to that recommended by the WHO (58). Because the country's current cardiovascular health is compromised, actions and strategies to reduce sodium consumption among the population are urgently needed to improve morbidity and mortality in Mexico.

## Author Contributions

IC-N and JV developed the draft of the article. CN and AA carried out a miniature review on the challenges of the main problem. IC-N, JV, and SB wrote the study's recommendations and conclusions. All authors contributed to the article and approved the submitted version.

## Funding

This research was funded by Bloomberg Philanthropies (ID: 019-71206).

## Conflict of Interest

The authors declare that the research was conducted in the absence of any commercial or financial relationships that could be construed as a potential conflict of interest.

## Publisher's Note

All claims expressed in this article are solely those of the authors and do not necessarily represent those of their affiliated organizations, or those of the publisher, the editors and the reviewers. Any product that may be evaluated in this article, or claim that may be made by its manufacturer, is not guaranteed or endorsed by the publisher.
